# Immune spleen cells attenuate the inflammatory profile of the mesenteric perivascular adipose tissue in obese mice

**DOI:** 10.1038/s41598-021-90600-0

**Published:** 2021-05-27

**Authors:** Renée de Nazaré Oliveira da Silva, Rosangela Aparecida Santos-Eichler, Carolina Dias, Stephen Fernandes Rodrigues, Dominik S. Skiba, Richardt Gama Landgraf, Maria Helena Catelli de Carvalho, Tomasz Guzik, Ricardo Ambrósio Fock, Eliana Hiromi Akamine

**Affiliations:** 1 Department of Pharmacology, Institute of Biomedical Sciences, University of São Paulo, São Paulo, Brazil; 2 Department of Clinical and Toxicological Analysis, School of Pharmaceutical Sciences, University of São Paulo, São Paulo, Brazil; 3 Institute of Cardiovascular and Medical Sciences, University of Glasgow, Glasgow, UK; 4 Department of Pharmaceutical Sciences, Federal University of São Paulo, Diadema, Brazil; 5 Department of Experimental Genomics, Institute of Genetics and Animal Biotechnology Polish Academy of Sciences, Jastrzebiec, Poland

**Keywords:** Obesity, Obesity

## Abstract

The perivascular adipose tissue (PVAT) differs from other fat depots and exerts a paracrine action on the vasculature. The spleen has an important role in the immune response, and it was observed to have either a protective role or a contribution to obesity-related diseases. However, the relation between spleen and PVAT is elusive in obesity. We investigated the role of spleen in the inflammatory profile of the mesenteric PVAT (mPVAT) from mice fed a high-fat diet (HFD) for 16 weeks. Male C57Bl/6 mice were sham-operated or splenectomized (SPX) and fed a HFD for 16 weeks. mPVAT morphology was evaluated by hematoxylin and eosin staining, infiltrated immune cells were evaluated by flow cytometry, inflammatory cytokines were evaluated by ELISA and the splenic cell chemotaxis mediated by mPVAT was evaluated using a transwell assay. In SPX mice, HFD induced adipocyte hypertrophy and increased immune cell infiltration and proinflammatory cytokine levels in mPVAT. However, none of these effects were observed in mPVAT from sham-operated mice. Spleen from HFD fed mice presented reduced total leukocytes and increased inflammatory markers when compared to the spleen from control mice. Chemotaxis of spleen cells mediated by mPVAT of HFD fed mice was reduced in relation to standard diet fed mice. The spleen protects mPVAT against the effects of 16-week HFD. This information was missing, and it is important because PVAT is different from other fat depots and data cannot be extrapolated from any type of adipose tissue to PVAT.

## Introduction

It is well established in obesity that the white adipose tissue (WAT), mainly from the visceral depot, presents an increase in inflammatory markers and infiltration of immune cells, as well as hypertrophy of adipocytes^1^. The perivascular adipose tissue (PVAT) has been considered to be a fourth type of adipose tissue^2^ and it has been reported to modulate not only the vascular tonus, but also inflammation, mainly due to secretion of some substances facilitated by its proximity to the blood vessels^3^. Some studies have demonstrated that PVAT from obese humans presents increased levels of inflammatory markers such as monocyte chemoattractant protein-1 (MCP-1), interleukin (IL)-6 and IL-8^4,5^. The increased chemokine and proinflammatory cytokine secretion from PVAT leads to a mobilization of immune cells, mainly macrophages and lymphocytes^6^.

The spleen is one of the most important lymphoid tissues in the body and it is a complex organ with a highly organized compartmentalization and an intricate microcirculatory system. A number of processes occur in the red pulp, namely blood filtration, old or damaged red blood cell phagocytosis, iron recycling, extramedullary hematopoiesis and fast release of antibodies produced by plasmablasts. In the spleen of mice, the white pulp is arranged along the central arteries and consists of T cells organized in periarteriolar lymph sheath and B cells organized in follicles surrounded by a marginal zone, located at the interface of the white and the red pulp. The latter is composed of specialized populations of macrophages, resident B cells, dendritic cells and T cells. Both cellular and humoral immunity get started in the white pulp^7,8^. Therefore, the spleen plays an important role in regulating immune homoeostasis.

A protective role of spleen has been reported against inflammation of WAT and obesity-related diseases, such as steatohepatitis, non-alcoholic fatty pancreas disease, insulin resistance and chronic kidney disease, which may have the participation of B cells and IL-10^9–12^. In contrast, another study, using an induced-obesity model with monosodium glutamate, showed that splenectomy reduces hypertrophy of adipocytes and insulin resistance, indicating a contribution of spleen in the progression of obesity^13^. However, the relation between spleen and PVAT is elusive in obesity. Thus, the aim of the present study was to evaluate the role of the spleen in the inflammatory profile of the mesenteric PVAT (mPVAT) of mice fed with a high-fat diet (HFD).

## Results

### Characterization of obesity

Since both worsening and improvement of metabolic parameters have been shown by splenectomy in obese animals^9–13^, we first analyzed the effects of HFD feeding for 16 weeks on the adiposity, glucose homeostasis and circulant adiponectin in mice with and without the spleen.

**Figure 1:**
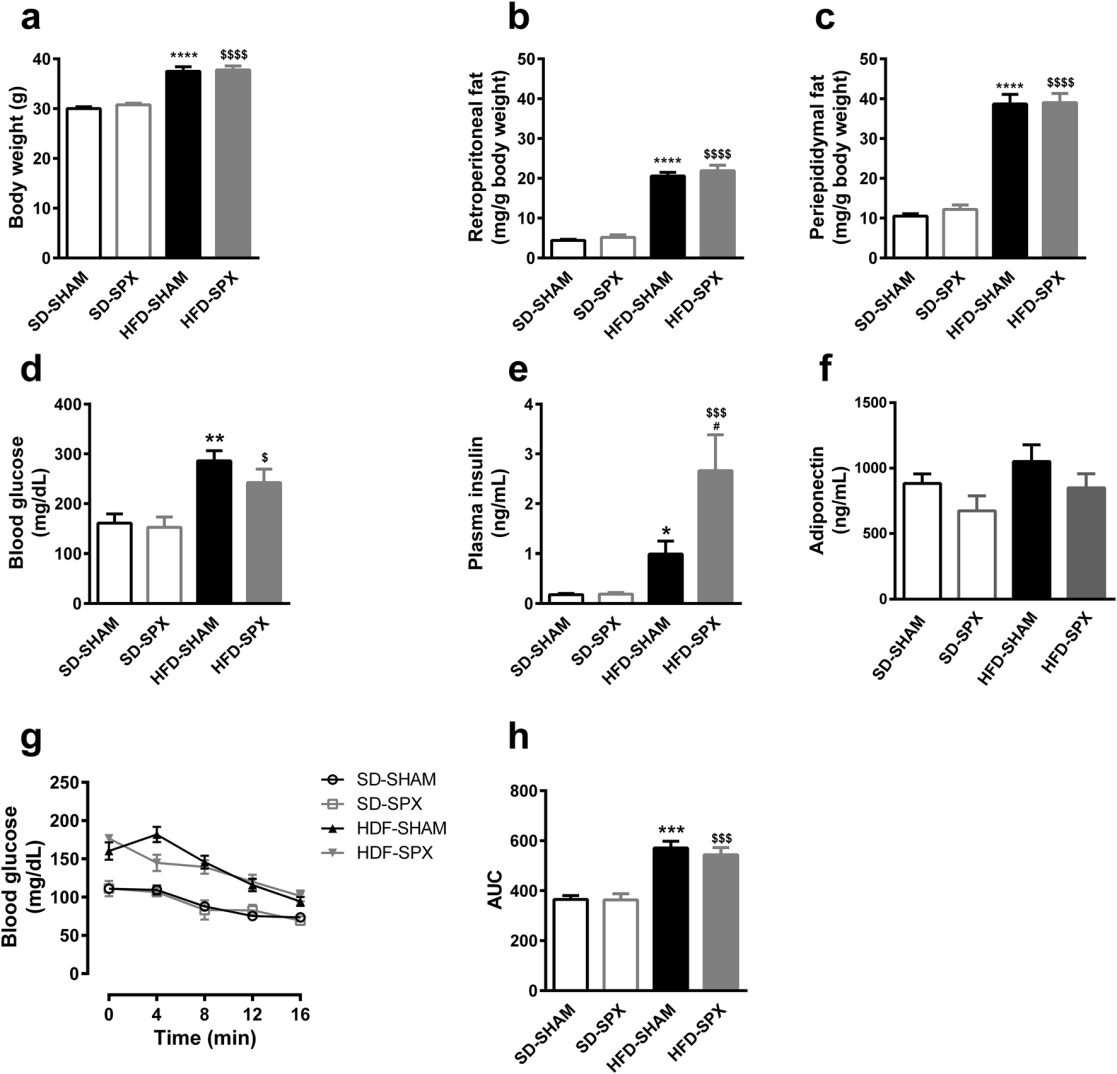
High-fat diet (HFD) increased adiposity and impaired glucose homeostasis in the presence and absence of the spleen. (**a**) Final body weight, (**b**) retroperitoneal and (**c**) periepididymal fat mass, (**d**) blood glucose, (**e**) plasma insulin and (**f**) plasma adiponectin levels, (**g**) blood glucose levels and area under the curve (AUC) during insulin tolerance test (ITT) in sham-operated (SHAM) or splenectomized (SPX) mice fed with standard diet (SD) or HFD. (n = 32 to 33 per group and n = 5 to 8 per group for blood glucose, insulin and adiponectin levels and ITT). Data were expressed as mean ± SEM. ANOVA: **P < 0.01 and ****P < 0.0001, HFD-SHAM versus SD-SHAM; ^$$$^*P* < 0.001 and ^$$$$^*P* < 0.0001, HFD-SPX versus SD-SPX.

HFD feeding for 16 weeks promoted an increase in body weight, periepididymal and retroperitoneal fat pads, blood glucose levels, plasma insulin levels and area under the curve of glucose concentration during insulin tolerance test in SHAM and splenectomized (SPX) mice in comparison to standard diet feeding (Fig. 1). Plasma insulin concentration was higher in SPX than in SHAM mice fed with HFD, but none of other parameters was found to be significantly different between SPX and SHAM mice receiving the same diet (Fig. 1). Plasma adiponectin levels were similar among the groups (Fig. 1).

### Effects of 16-week HFD feeding and splenectomy on the morphology, inflammatory cells and cytokine levels in mPVAT

The next step was the evaluation of how splenectomy impacts the effects mediated by 16-week HFD feeding on the histological features of mPVAT.

**Figure 2:**
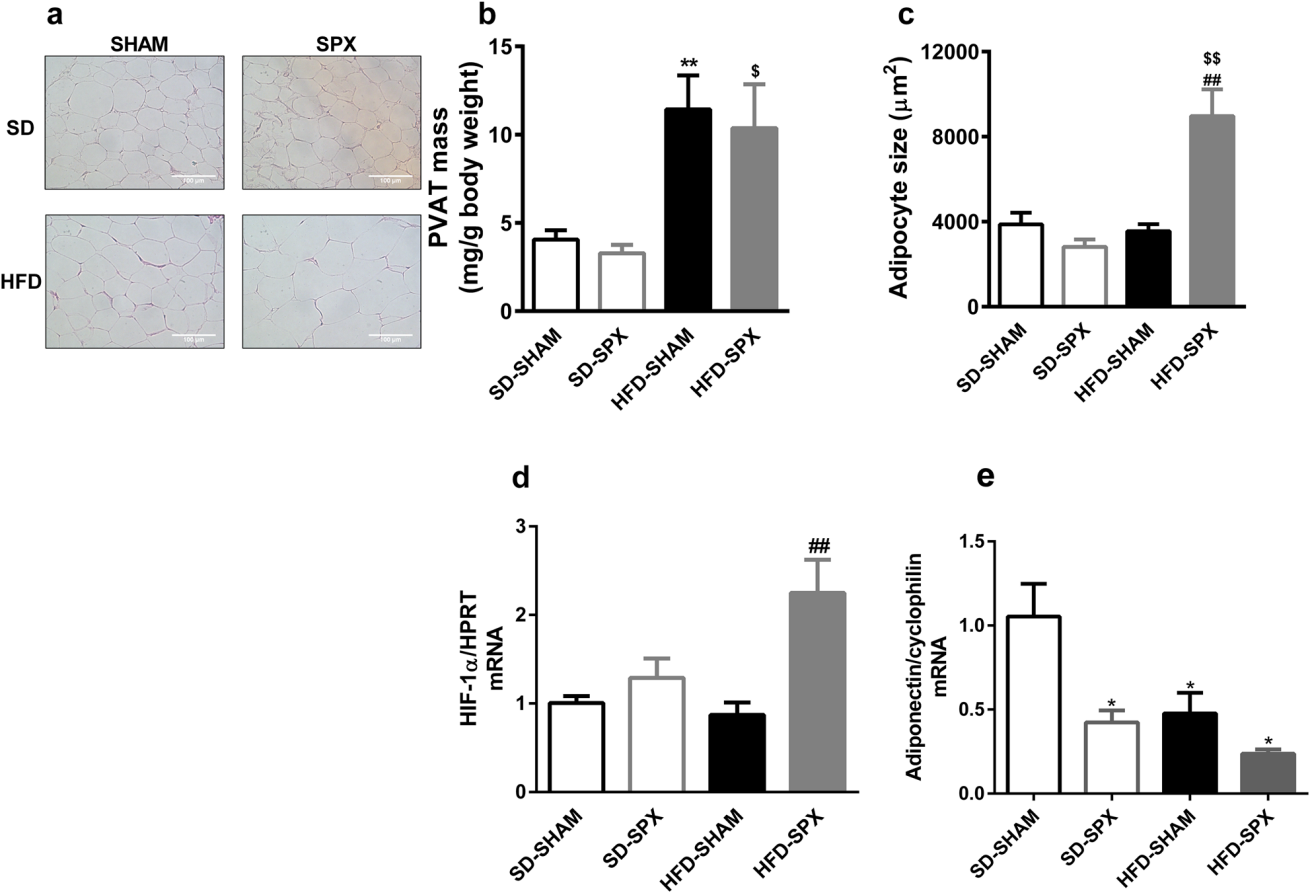
Splenectomy promoted adipocyte hypertrophy in mesenteric PVAT (mPVAT) of mice fed a high-fat diet (HFD). (**a**) Representative histological images, (**b**) mass, (**c**) adipocyte size, (**d**) HIF-1 α mRNA levels and (**e**) adiponectin mRNA levels in mPVAT from sham-operated (SHAM) or splenectomized (SPX) mice fed with standard diet (SD) or HFD. (n = 4 per group and n = 8 to 10 per group for PVAT mass). Data were expressed as mean ± SEM. ANOVA: *P < 0.05 and **P < 0.01, SD-SPX and HFD-SHAM versus SD-SHAM; ^$^*P* < 0.05 and ^$$^*P* < 0.01, HFD-SPX versus SD-SPX; ^##^*P* < 0.01, HFD-SPX versus HFD-SHAM.

The mass of mPVAT was increased in all mice fed 16-week HFD (Fig. 2b). However, at that time hypertrophy was still not observed in that tissue in response to HFD in SHAM mice. On the other hand, in the PVAT from SPX mice on HFD we observed hypertrophy. (Fig. 2a,c). Since hypoxia develops with adipocyte hypertrophy and hypoxia triggers hypoxia-inducible factor (HIF)-1 α transcription^14^, we evaluated the mRNA content of this factor in mPVAT. HIF-1 α mRNA levels were found increased only in mPVAT from SPX on HFD when compared to SHAM on either standard or HFD (Fig. 2d). This is in accordance with the hypertrophy observed only for SPX mice on HFD. Thus, we evaluated the impact of splenectomy on the mRNA expression of adiponectin because it is an adipose tissue-derived anti-inflammatory factor and it is reduced in adipose inflammation^1^. Adiponectin mRNA expression was reduced in mPVAT from SPX on standard diet and from both SHAM and SPX on HFD in comparison to SHAM on standard diet (Fig. 2e).

**Figure 3:**
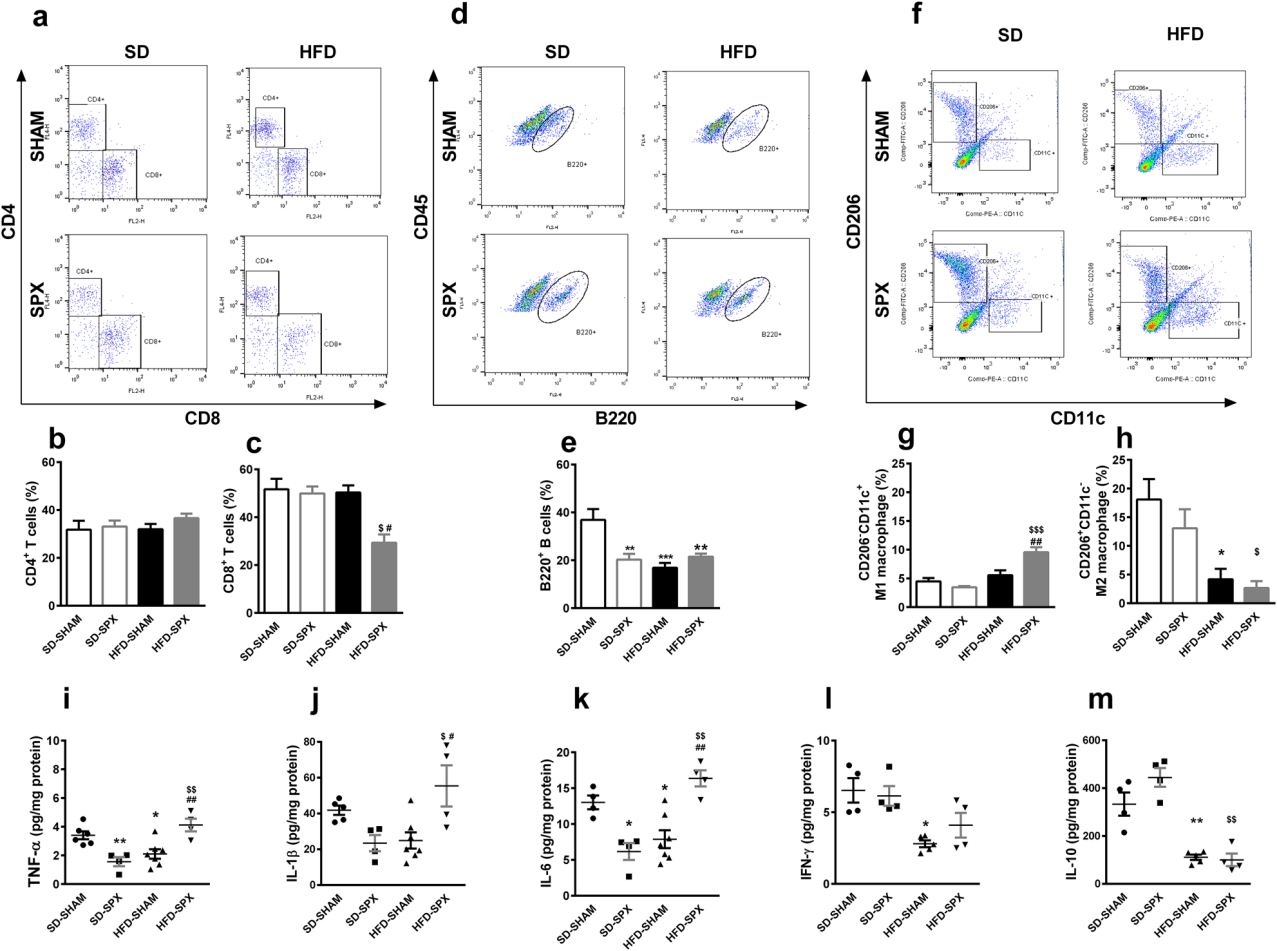
Splenectomy increased inflammatory markers in mesenteric PVAT (mPVAT) of mice fed a high-fat diet (HFD). (**a**, **d**, **f**) Representative dot plots of flow cytometry and populations of (**b**) CD4^+^ and (**c**) CD8^+^ T lymphocytes (percentage of CD3^+^ cells), (**e**) B220^+^ B lymphocytes (percentage of CD45^+^ cells) and (**g**) CD206^-^CD11c^+^ (M1) and (**h**) CD206^+^CD11c^−^ (M2) macrophages (percentage of F4/80^+^ cells) in the mPVAT of sham-operated (SHAM) or splenectomized (SPX) mice fed with standard diet (SD) or HFD. Results from two different experiments (n = 5 to 9 per group). (**i**–**m**) Cytokine levels in mPVAT of SD-SHAM, SD-SPX, HFD-SHAM and HFD-SPX mice. (n = 5 to 7 per group). Data were expressed as mean ± SEM. ANOVA: *P < 0.05, **P < 0.01 and ***P < 0.001, SD-SPX and HFD-SHAM versus SD-SHAM; ^$^*P* < 0.05, ^$$^*P* < 0.01, ^$$$^*P* < 0.001 and ^$$$$^*P* < 0.0001, HFD-SPX versus SD-SPX; ^#^*P* < 0.05 and ^##^*P* < 0.01, HFD-SPX versus HFD-SHAM.

We then aimed to determine the role of spleen in the profile of the population of immune cells in mPVAT under HFD, analyzing the immunophenotyping of cells by flow cytometry. The representative dot plots of flow cytometry are shown in Fig. 3a,d,f.

In mPVAT from SHAM mice, the percentage of the population of CD4^+^ and CD8^+^ T lymphocytes and M1 macrophages were not affected by 16-week HFD (Fig. 3b,c,g). However, the percentage of the population of M2 macrophages was reduced in PVAT from SHAM mice on HFD (Fig. 3e), as well as the percentage and absolute numbers of B220^+^ B lymphocytes (Fig. 3e and Supplementary Fig. 5S online).

Additionally, mPVAT of SPX mice fed with the standard diet presented a reduction in the percentage (Fig. 3e) and absolute number (Supplementary Fig. 5S online) of B220^+^ B lymphocytes in comparison to SHAM animals fed with the same standard diet. In relation to the other cell populations studied, no differences were observed when comparing those two groups (Fig. 3 and Supplementary Fig. 5S online).

Interesting results were found in the PVAT of SPX mice fed with HFD, in which a reduced percentage and absolute numbers of CD8^+^ cells were observed in relation to SHAM mice fed with the same diet (Fig. 3c and Supplementary Fig. 5S online). However, splenectomy did not further change the reduction in B220^+^ B lymphocytes and M2 macrophages observed in SHAM mice fed with HFD (Fig. 3e,h and Supplementary Fig. 5S online). In addition, an increased percentage and absolute numbers of M1 macrophages were observed in PVAT of SPX fed with HFD in comparison to the other groups (Fig. 3g and Supplementary Fig. 5S online).

In order to further evaluate the impact of the splenectomy in the inflammatory profile of mPVAT of mice under HFD, we analyzed the cytokine levels. Regarding cytokines derived from mPVAT, the levels of tumor necrosis factor (TNF)-α, IL-1β, IL-6, interferon (IFN)-γ and IL-10 were all reduced in PVAT of SHAM mice fed 16-week HFD in comparison to SHAM mice fed with a standard diet (Fig. 3i–m). Additionally, splenectomy in mice fed with the standard diet also reduced the TNF-α, IL-1β and IL-6 levels, without changing the IFN-γ and IL-10 levels in comparison to SHAM mice fed with the same diet (Fig. 3i–m). In contrast, TNF-α, IL-1β and IL-6 levels were increased in mPVAT of SPX mice fed with HFD in comparison to SHAM mice fed with the same diet and SPX mice fed with the standard diet (Fig. 3i–k). On the other hand, IFN-γ and IL-10 levels were differentially regulated in SPX mice fed HFD. IFN-γ levels were higher than in SHAM mice fed with the same diet and similar to SPX mice fed with the standard diet, whereas IL-10 levels were similar to those in SHAM mice fed HFD and lower than in SPX mice fed with the standard diet (Fig. 3l,m).

In the present study, we did not aim to compare mPVAT to other fat pads. However, some parameters were evaluated also in periepididymal fat pad, which are presented in the Supplementary Fig. S1 online. Whereas periepididymal fat presented a different response to HFD for some parameters, the impact of splenectomy was similar in periepididymal fat and mPVAT. Differently from mPVAT, adipocytes were already hypertrophied and expression of adiponectin mRNA and percentage of M1 macrophages were increased in periepididymal fat of HFD-SHAM mice. Splenectomy further increased the size of adipocytes, maintained the increase in percentage of M1 macrophages and reduced the percentage of M2 macrophages and the adiponectin mRNA content in periepididymal fat (Supplementary Fig. S1a and S1b online).

### Splenic cell chemotaxis mediated by mesenteric PVAT (mPVAT)

**Figure 4:**
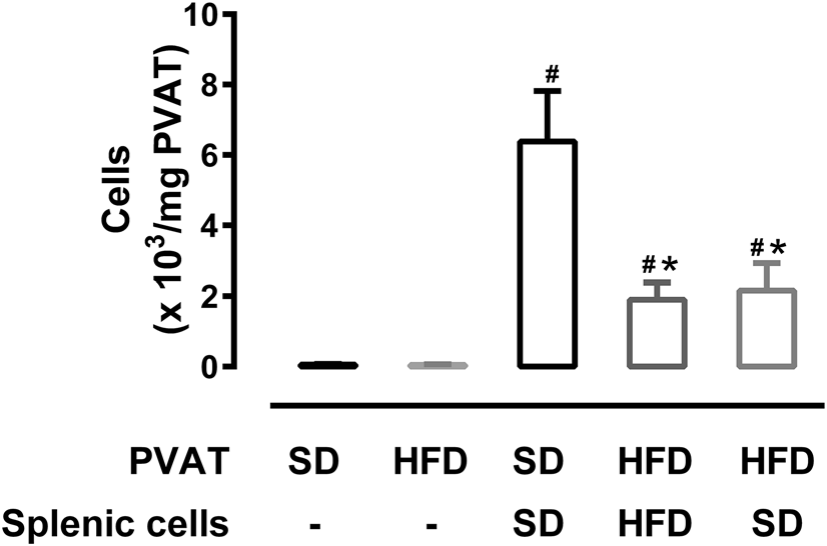
Splenic cell chemotaxis mediated by mesenteric PVAT (mPVAT) was reduced in mice fed a high-fat diet (HFD). Migration of splenic cells from SHAM mice fed with standard diet (SD) or HFD towards their respective mPVAT. Another set of experiments was performed with PVAT from mice fed with HFD using spleen cells from mice fed with SD. Control conditions involved placing only PVAT in the lower compartment. Results from two different experiments (n = 6 to 9 per group). Data were expressed as mean ± SEM. ANOVA: *P < 0.05, versus SD.

As an attempt to clarify the participation of the spleen in the immune cell infiltration in mPVAT, we evaluated the potential of mPVAT in mobilizing the splenic cells by using a transwell apparatus. All experimental conditions (when splenic cells were placed in the upper compartment and PVAT was placed in the lower compartment of the transwell) presented increases in transmigrated cells in relation to the control conditions (when only PVAT was placed in the lower compartment of the transwell). For mice fed with standard diet, approximately 5.8 × 10^3^ splenic cells migrated for each mg of PVAT. The migration of splenic cells towards mPVAT from mice fed HFD was found to be approximately 3 times lower when compared to control conditions (Fig. 4). In order to discard any deficiency in the cells derived from the spleen, potentially caused by HFD, we used splenic cells from mice fed with the standard diet to observe the migration of cells to mPVAT of mice fed with HFD. Our results demonstrated a reduced migration of those cells towards mPVAT (Fig. 4).

### Effects of HFD feeding on cellularity and inflammatory cytokines of spleen

**Figure 5:**
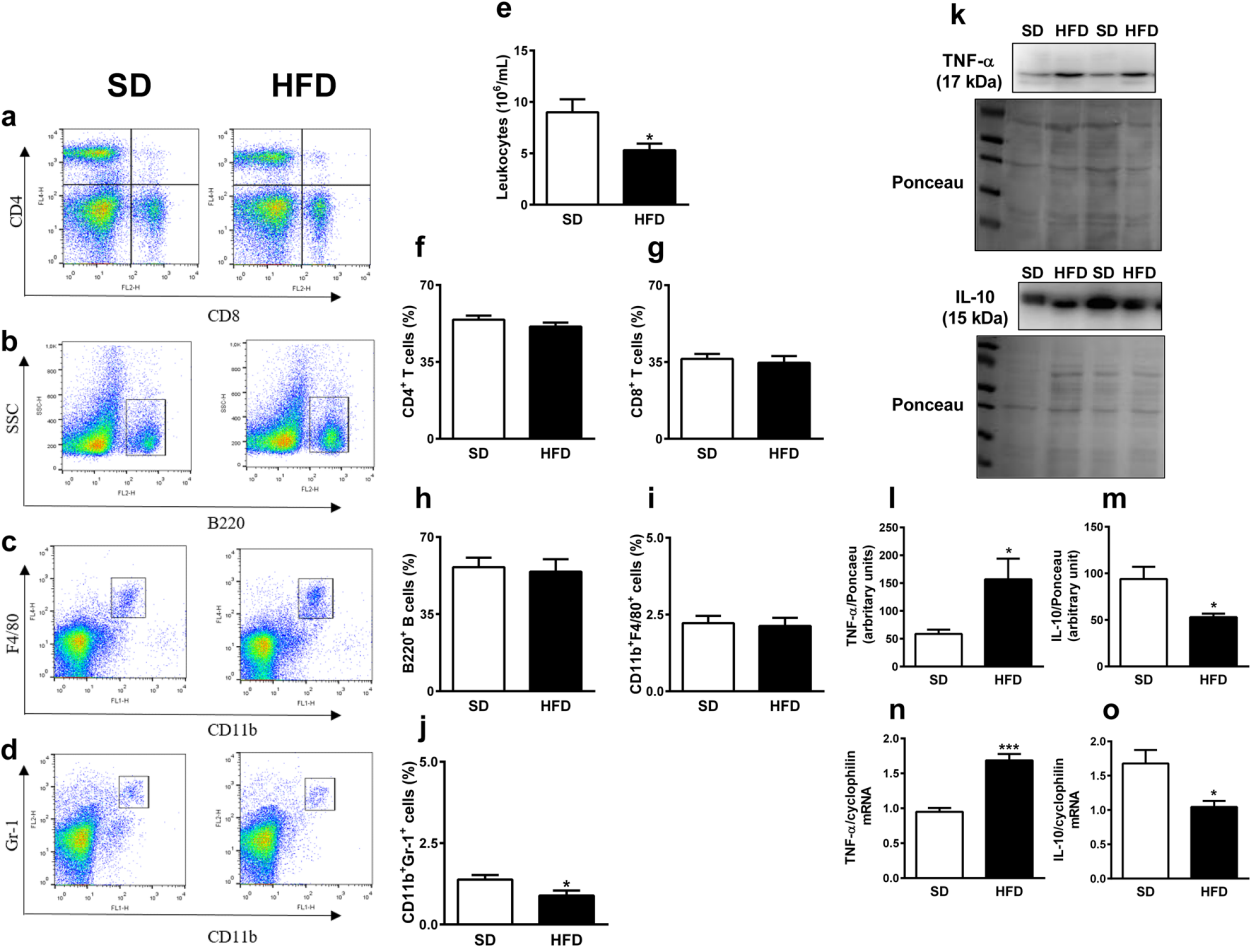
Mice fed a high-fat diet (HFD) presented leukopenia and increase in inflammatory markers in the spleen. (**a**–**d**) Representative dot plots of flow cytometry, (**e**) number of total leukocytes and populations of (**f**) CD4^+^ and (**g**) CD8^+^ T lymphocytes (percentage of CD3^+^ cells), (**h**) B220^+^ B lymphocytes, (**i**) CD11b^+^F4/80^+^ macrophages and (**j**) CD11b^+^Gr-1^+^ granulocytes (percentage of total cells) in the spleen of mice fed standard diet (SD) or HFD. Results from two different experiments (n = 5 to 8 per group). (**k**–**m**) Cropped blots, membranes stained with Ponceau and densitometry analysis for TNF-α and IL-10 in spleen of mice fed SD and HFD. (n = 4 per group). Full-length blots are shown in Supplementary Fig. S2. (**n**–**o**) TNF-α and IL-10 mRNA levels in spleen of mice fed SD and HFD. (n = 5 per group). Data were expressed as mean ± SEM. Unpaired *t*-test: *P < 0.05, HFD versus SD.

Finally, we evaluated the impact of a HFD in the populations of cells of the spleen. To do this, we immunophenotyped the dissociated spleen cells by flow cytometry. The representative dot plots of flow cytometry are shown in Fig. 5a–d.

The number of leukocytes in spleen of mice fed with HFD was reduced in comparison to mice fed with the standard diet (Fig. 5e), reducing the absolute numbers of different spleen cell populations (Supplementary Fig. S6 online). However, the percentage of splenic populations of CD4^+^ and CD8^+^ T lymphocytes (Fig. 5a,f,g), B220^+^ B lymphocytes (Fig. 5b,h) and CD11b^+^F4/80^+^ macrophages (Fig. 5c,i) were similar between mice fed with HFD and standard diet. However, the population of CD11b^+^Gr-1^+^ granulocytes was reduced in the spleen of mice fed HFD in comparison to mice fed the standard diet (Fig. 5d,j). The splenic protein and mRNA expression of TNF-α were higher and those parameters for IL-10 were lower in HFD mice than in control mice (Fig. 5k–o).

## Discussion

Spleen-derived cells may contribute to the inflammation of visceral WAT and progression of obesity^13^, although their protective role in obesity-related diseases has also been described^9,10,12^. Obesity has also an impact in the PVAT; however, the relation between PVAT and spleen is still unknown. Since PVAT is also a target of obesity, we investigated the possible role of spleen in the inflammatory profile of the mPVAT induced by HFD in mice. In the present study, we extend observations that spleen removal increase the concentration of adipokines and the infiltration of immune cells in the visceral WAT^10,11^, demonstrating a protective role of spleen against the effects of HFD on mPVAT.

We described here that 16-week on HFD in mice caused obesity, including an increase in the mass of mPVAT in all groups. In SHAM mice fed with HFD, the increased mass of mPVAT might be due to an increase in the recruitment of smaller cells, which serve as a potential reservoir for mature adipocytes^15^, since there were no changes in adipocyte sizes after 16 weeks of HFD feeding. In contrast, the increased mPVAT mass of SPX mice on HFD was associated with hypertrophy. In visceral WAT depots, we observed hypertrophy of adipocytes from SHAM mice on HFD and, in the absence of spleen, the visceral WAT presented an even larger size (Supplementary Fig. S1 online). Our results are in contrast to those found in the study of Gotoh et al.^10^. In that study, splenectomy reduced the lipid accumulation in adipocytes of periepididymal fat, whereas it accelerated lipid accumulation in liver in mice under 8-week feeding HFD. Despite we have not evaluated the liver, it is possible to hypothesize that in our model there was a disruption in the synchronized work between adipose tissue and liver, resulting in hypertrophic adipocytes. Since adipocyte size rather than number is an important marker of obesity-related diseases, our data indicate a protective role of spleen cells against the damage caused by HFD.

In agreement to the absence of adipocyte hypertrophy, we did not observe any increase in inflammatory factors (TNF-α, IL-1 β, IL-6) in mPVAT of SHAM mice on HFD, showing that 16-week HFD used in the present study promoted fat accumulation, but still without an inflammatory process. Nevertheless, TNF-α, IL-1β and IL-6 levels were increased along with higher HIF-1α mRNA levels in mPVAT of SPX mice on HFD, which presented hypertrophic adipocytes. These data reinforce the protective role of spleen against the effects of HFD on mPVAT. Also, this is indicative that the tissue is undergoing some sort of hypoxia, since the increase of those cytokines is a typical response to hypoxia, at least in WAT^16^. However, since HIF-1α is also post-translationally regulated, further investigation is needed to confirm the hypoxia in mPVAT of SPX mice on HFD.

An anti-inflammatory role is generally attributed to adiponectin^17,18^. Since absence of the spleen promoted an increase in inflammatory markers, an alteration in plasma adiponectin levels would be expected in splenectomized mice fed HFD, but no change was observed in those levels. This finding is different from that of Gotoh et al.’s study, in which splenectomy promoted a reduction of serum adiponectin^10^. It is possible that differential changes in adiponectin production might be observed in each adipose tissue depot at 16 weeks of HFD feeding and splenectomy surgery, since there were differences in mRNA expression of adiponectin in mPVAT and periepididymal fat pad (Supplementary Fig. S1 online). The circulant adiponectin levels represent the resultant sum of the overall released adiponectin.

It is well known that the resident macrophages in the fat depots, both visceral WAT and PVAT, undergo macrophage polarization to M1 after an inflammatory stimulus, acquiring a macrophage proinflammatory phenotype and contributing to tissue inflammation^19^. Although we observed a reduction in M2 macrophage infiltration in mPVAT, we could not observe any effect of the HFD on M1 macrophage infiltration in mPVAT from SHAM mice. Nevertheless, we have observed increased M1 macrophage infiltration only in visceral adipose tissue compared to mice fed with HFD, whereas M2 macrophage infiltration was not changed (Supplementary Fig. S1 online). Those results show that HFD alone promoted only a slight inflammation, dependent on the fat pad. Our results were different from data from another study that showed an increase in M1 macrophage infiltration in mPVAT in mice fed a HFD^20^, but it is possible that macrophage infiltration is a response to PVAT inflammation^21^. In SPX mice fed with HFD, on the other hand, besides an increase in pro-inflammatory cytokines we observed an increase of M1 macrophages and a reduction of M2 macrophages, although the latter effect seems to be due to HFD rather than lack of the spleen.

Alder et al. (2014) have demonstrated that CD8^+^ T lymphocytes are increased in the visceral WAT of mice submitted to HFD for 3 weeks^22^. Nishimura et al. (2009) also reported an increase in CD8^+^ T cells, in response to 16-week HFD, considering them as the main factor that precedes and contributes to M1 macrophage accumulation in visceral WAT in obesity^23^. However, in the present study, we did not observe changes in the percentage of CD4^+^ and CD8^+^ T cells in PVAT of SHAM mice on HFD. It has been shown that one type of CD8^+^ T cells (regulatory) can secrete inhibitory cytokines, such as IL-10, keeping the tissue in an inflammation-free state^24^. Since in mPVAT of HFD-SHAM mice the unaltered CD8^+^ T cells were associated to reduced pro-inflammatory cytokine levels, but, in mPVAT of HFD-SPX, reduced CD8^+^ T cells were associated to increased pro-inflammatory cytokynes, it should be investigated in future studies whether CD8^+^ T cells play an anti-inflammatory role in mPVAT.

Despite the spleen is a reservoir for a variety of immune cells, B cells are present in the highest proportion than other cells^25^. Thus, splenectomy could have a major effect on the quantity of such cells. In fact, in non-obese individuals, splenectomy promotes a deficiency in B cells^26–28^, whereas non-obese splenectomized mice displayed no changes in CD8^+^ cell numbers^29^. A cellular mechanism seems to be involved in the protective role of spleen against the HFD-induced effects. It has been described that IL-10-producing B cells from spleen protect visceral WAT against inflammation induced by HFD^10^. Spleen supports a pool of IL-10-producing B cells, which act in WAT and that, with a dietary lipid excess, are expanded^12^. However, the demand for IL-10-producing B cells with the expansion of WAT exceeds the B cell capacity of supplying this cytokine, causing a reduction in the protective action of IL-10^12^. This effect exacerbates M1 macrophage infiltration and obesity-related disorders^30^.

In the present study, despite the fact that the spleen has played a protective role in mPVAT of mice fed HFD, we could not see an association between lack of spleen and reduced B cells (in mice fed HFD) and between reduced B cell population and reduced IL-10 levels in mPVAT (in mice fed standard diet). This suggests a different source of IL-10, besides splenic cells in mPVAT, and that HFD alters the non-splenic pool of B cells. IL-10 may also be produced by visceral WAT-derived M2 macrophages, which is inhibited by free fatty acids^31^. Thus, it is possible that reduction in IL-10 levels was due to the reduction in M2 macrophages in mPVAT in mice fed HFD (SHAM and SPX). Moreover, a non-splenic pool of B cells could involve, for example, the adipose natural regulatory B cells found in subcutaneous and visceral WAT, which constitutively produce IL-10 and negatively control WAT inflammation^32^. Taken together, those findings suggest that cells distinct from splenic B cell pool may be responsible for IL-10 levels in mPVAT, which were impaired by HFD.

We also evaluated the HFD impact in the spleen. The expression of TNF-α was increased and IL-10 was reduced in the spleen from mice fed HFD for 16 weeks, indicating a local inflammatory process. A possible explanation for our results is an unbalance in splenic redox status, since production of pro-inflammatory cytokines by the spleen was increased in HFD-induced obese mice and was associated with a reduced antioxidant capacity and oxidative stress^33^. However, Gotoh et al. (2012)^10^ observed that 8-week HFD feeding reduced the levels of TNF-α, IL-1β and MCP-1 in the spleen, suggesting that the diet time influences the spleen inflammation process. However, there is another difference beyond the duration of diet treatment, represented by the albumin supplementation in that study. Nevertheless, splenectomy of mice fed an HFD increased those factors in WAT and liver^10^. It is interesting to note that independently of the spleen inflammation, its removal promotes inflammation in adipose tissue (visceral WAT and mPVAT) and liver.

In order to evaluate the communication, through chemotaxis, between spleen cells and mPVAT in response to HFD, we assessed the splenic cell migration to mPVAT. We observed a reduced splenic cell migration to mPVAT from HFD fed mice, suggesting a poor chemotaxis of spleen leukocytes. It could be pointed out that the size of the spleen cells may increase under stimulation^34^, for example by HDF, and that the larger cells could not migrate through the membrane used in the study. However, when the chemotaxis assay was performed using splenic cells from mice fed with standard diet, we still observed a decrease in number of migrated cells toward mPVAT from HDF fed mice. A limitation of the present study is that the transmigrated splenic cells toward mPVAT were not identified and further studies should evaluate if HFD induces a global decrease in capacity of mPVAT to recruit splenic cells or if it decreases the recruitment of a specific type of cell. It is interesting that the spleen had an apparent anti-inflammatory role, and splenectomy promoted a proinflammatory status in mPVAT. Taken together, these results demonstrate a similar protective role of spleen in mPVAT and other depots, even if PVAT represents a different kind of adipose tissue^2^.

It is important to note that, in the present study, 16-week HFD was not sufficient to promote adipocyte hypertrophy and promoted minimal changes of inflammatory markers in mPVAT. However, an increase in TNF-α concentration has been observed in the mPVAT from 16-week HFD fed rats^20^ and in the abdominal PVAT of 16-week HFD fed mice, but not in the aortic PVAT^35^. This suggests that the susceptibility to HFD-induced inflammation depends on the animal model and the anatomical location of PVAT. Moreover, this difference may be explained by the fact that the C57Bl/6 mice used here do not have a mutation in the nicotinamide nucleotide transhydrogenase gene. It has been reported that C57Bl/6 J substrains that present this mutation display a higher susceptibility to gain fat mass and to an impairment of glucose tolerance induced by HFD in comparison to C57Bl/6 without the mutation^36,37^.

Our results demonstrate, for the first time, that the spleen plays a role preventing adipocyte hypertrophy and inflammation response of mPVAT from mice fed with 16-week HFD. In conclusion, our findings show a role of spleen in modulating the inflammatory state in mPVAT from 16-week HFD fed mice, and that the modulation is tissue specific among different fat depots. Considering the importance of PVAT in the vascular regulation, both physiologically and in diseases, the lack of the spleen could lead to a disturbance of the mPVAT function with important consequences for blood vessels. Further studies should investigate the impact of the lack of the spleen on the anticontractile action mediated by mPVAT.

## Material and methods

### Ethical statement

The Ethics Committee of the Institute of Biomedical Sciences of University of São Paulo approved the protocols of the present study (number 90/2013). All procedures were performed in accordance with the Brazilian National Law for Use and Welfare of Experimental Animals (nº 11.794) and the rules issued by the National Council for Control and Animal Experimentation (CONCEA) and the ARRIVE guidelines^38^.

### Animal

Three-week old C57Bl/6 male mice were obtained from the breeding stock of the Facility for SPF Mice Production at University of São Paulo Medical School. Mice were housed in a temperature-controlled room (22 ± 2 °C), under a 12 h light/12 h dark cycle, and provided with food and water ad libitum.

### Splenectomy

Four-week old mice were anesthetized with isoflurane (5% for induction and 2% for maintenance in oxygen). After shaving and sterilization, the upper left abdominal quadrant was incised, and the spleen was carefully removed (SPX). Or else, the abdomen was incised, but the spleen was not removed, for the control procedure (SHAM).

### Induction of obesity

SPX and SHAM mice were divided in two groups and fed with standard diet (3.8 kcal/g: 70% carbohydrate; 20% protein; 10% fat) (Nuvilab CR1, Nuvital Nutrients S.A., Brazil) or with a high-fat diet (HFD) (5.4 kcal/g: 26% carbohydrate; 15% protein, 59% fat) (PragSoluções, Brazil) for 16 weeks. The body weight was recorded weekly, and the final weight after the 16-week diet period was presented.

### Insulin tolerance test (ITT)

Mice were fasted for 6 h before the ITT. Sample of blood was taken from tail vein and blood glucose was measured by using a monitor and testing striping (Accu-Check, La Roche, Brazil) before (time 0) and after 4, 8, 12 and 16 min of intraperitoneal administration of the solution of insulin (0.75 IU/kg of body mass).

### Blood collection, tissue preparation and blood glucose and insulin measurements

At the end of the diet period, mice were fasted for 6 h and anesthetized with 3% isoflurane. Blood was obtained by cardiac puncture and transferred to a container with EDTA (BD Biosciences). Mesentery and periepididymal and retroperitoneal fat pads were excised. The spleen from SHAM mice was excised for analysis. In order to isolate the mesenteric PVAT, the intestine was excised and pinned in Sylgard-clad bottom of a dish filled with a cold physiological salt solution. Under a dissection microscope, PVAT was removed along the branches of the mesenteric vessels (from first order arteries and the corresponding venules to the smallest vessels closer to the intestine), removing any lymph nodes and lymphatic vessels. mPVAT, periepididymal and retroperitoneal fat pads were weighed.

Blood samples were centrifugated (5000*g* for 15 min at 4 °C) to obtain plasma. Glucose levels were measured by using colorimetric kit (Labtest, Brazil). Insulin levels were determined by using a commercial ELISA kit (Cayman, USA).

### Histological analysis

mPVAT was fixed overnight in 10% PBS-buffered formalin and were thereafter stored in 70% ethanol. Fixed samples were paraffin-embedded and 5-μm thick sections were obtained from each sample, which were stained with hematoxylin and eosin. Sections were viewed under a microscope (Nikon, Japan) with a 20X objective. Photomicrographs were analyzed by using Image J (NIH, USA).

### Analysis of leukocytes in tissues

The spleen was passed through a 70 µm cell strainer (Corning, USA) and gently ground using the plunger of a syringe with 3 mL of PBS containing 1% fetal bovine serum to yield single-cell suspensions. Following centrifugation (400 g, 8 min), red cells were lysed using a hypotonic sodium chloride solution (0.2%), followed by incubation with a cold hypertonic sodium chloride solution (1.6%) for 30 s each. The cells were then centrifuged at 1500*g* for 6 min and the supernatant was discarded. Cells were stained with 0.01% Trypan blue and counted in a hemocytometer to obtain the total number of leukocytes. mPVAT was minced and digested using collagenase type XI (125 U/mL), collagenase type IS (450 U/mL) and hyaluronidase IV-S (60 U/mL) in Hank’s balanced salt solution with calcium and magnesium (Gibco, Thermo Fisher Scientific, USA) for 20 min, at 37 °C, shaking at 600 g. The digested tissues were passed through a 70 µm cell strainer (Corning, USA) and gently ground using the plunger of a syringe. Following centrifugation (400*g*, 8 min), the supernatant was discarded by inversion.

Single cell suspensions were then incubated with 0.1 μg/mL of each antibody for 20 min. The information on the antibodies is presented in the Supplementary Table S1 online. For immunophenotyping, PVAT cells were gated, and B lymphocytes were identified as CD45^+^ B220^+^; T lymphocytes were identified as CD3^+^ and CD4^+^ or CD3^+^ and CD8^+^. Additionally, macrophages were gated as F4/80^+^ and identified as M1 macrophages by F4/80^+^, CD206^−^ and CD11c^+^ or as M2 macrophages identified by F4/80^+^, CD206^+^ and CD11c^-^. The flow cytometry analysis strategy is described in Supplementary Figure S3 online. For immunophenotyping spleen cells, first the cells were gated and the population of B cells and T cells were identified. B cells were characterized as B220^+^ cells, and T cells were characterized as CD3^+^ and CD4^+^ or CD3^+^ and CD8^+^ cells. Additionally, immature myeloid cells were identified as CD11b^+^ and Gr-1^+^ and macrophages as F4/80^+^ and CD11b^+^. Once labelled, at least 1 × 10^4^ cells were acquired by flow cytometry. The flow cytometry analysis strategy is described in Supplementary Figure S4 online. To establish negative controls, we prepared unstained and stained cells with fluorescence-minus-one (FMO) control stain sets. Data were acquired on a FACS CantoII (FACScan®, BD Biosciences, USA), and FlowJo® 10 software (Tree Star Inc., USA) was used for data analysis.

### Quantitative real-time PCR

Total cellular RNA was isolated from mPVAT and spleen using TRIzol® Reagent (Invitrogen, Thermo Fisher Scientific, USA) according to the manufacturer’s instructions. DNase I was employed to digest DNA to obtain pure RNA prior to the reverse transcriptase reaction. Total RNA (2 μg) was used for first-strand cDNA synthesis using High Capacity cDNA kit (Thermo Fischer Scientific, USA). Twenty ng of cDNA samples were submitted to real-time PCR amplification using GoTaq® qPCR Master Mix (Promega, USA) and specific oligonucleotides for hypoxia-inducible factor (HIF)-1α (forward: AGTCAGCAACGTGGAAGGT; reverse: CGTCATGGGTGGTTTCTTG; 101 bp, NM_001313919.1) and adiponectin (forward: GAGAAAGGAGATGCAGGTCTTC; reverse: ACGCTGAGCGATACATAAG; 145 bp, NM_009605.5) for mPVAT and TNF-α (forward: ATG AGC ACA GAA AGC ATG ATC; reverse: TAC AGG CTT GTC ACT CGA ATT; 275 bp, NM_013693.2) and IL-10 (forward: AGGCGCTGTCATCGATTTCT; reverse: ATGGCCTTGTAGACACCTTGG; 104 bp, NM_010548.2) for spleen. Hypoxanthine guanine phosphoribosyl transferase (HPRT) (forward: TGCTGACCTGCTGGATTACA; reverse: TTTATGTCCCCCGTTGACTGA; 120 bp, NM_013556.2) and cyclophilin A (forward: TATCTGCACTGCCAAGACTGAGT; reverse: CTTCTTGCTGGTCTTGCCATTCC; 127 bp, NM_008907.2) were used as internal controls. Real-time PCR reactions were performed using the Corbett Research system (Corbett Life Sciences, Australia). The conditions for PCR were as follows: 95 °C for 2 min, then 40 cycles of 95 °C for 15 s and 60 °C for 1 min. Expression data were calculated from the cycle threshold (Ct) value using the ΔΔCt method for quantification^39^. Expression of HPRT and cyclophilin A mRNA was used for normalization. Results were expressed as fold increase compared to control.

### Western blot

The spleen was homogenized in RIPA buffer (Millipore, USA) containing 1 μg/µL of a protease inhibitor cocktail and total protein concentration was determined by the BCA method (Thermo Fisher Scientific, USA). Samples were treated with Laemmli’s buffer containing 350 mM dithiothreitol. Fifty µg of total protein were resolved by sodium dodecyl-sulfate polyacrylamide gel electrophoresis, transferred to polyvinylidene fluoride membrane (Amersham Hybond-P, GE Healthcare Life Sciences, UK) and incubated overnight at 4 °C with antibodies anti-IL-10 (1:1000; Novus Biologicals, JES5-2A5, USA) and anti-tumor necrosis factor (TNF)-α (1:500, BioLegend, 506101, USA) diluted in blocking solution (3% bovine serum albumin in TBS-T solution: 10 mM Tris, 150 mM NaCl and 0.02% Tween 20). Membranes were then washed with TBS-T solution and incubated with anti-rat IgG (1:2000, Abcam, USA, for IL-10) and anti-hamster IgG (1:2000, Jackson Immuno Research, USA, for TNF-α) antibodies conjugated to horseradish peroxidase diluted in blocking solution. Chemiluminescence signal was detected by an image system (Gel Logic, Carestream Molecular Imaging, USA) and the intensity of the bands was quantified by optical densitometry through the use of the ImageJ software (NIH, USA). Bands obtained from spleen were normalized by densitometry of their respective line bands stained with Ponceau S. All values were expressed as arbitrary units (UA).

### ELISA

mPVAT was homogenized in RIPA buffer (Millipore, USA) containing 1 µg/µL of a protease inhibitor cocktail and total protein concentration was determined by the BCA method (Thermo Fisher Scientific, USA). The concentrations of TNF-α, IL-1β, interferon (IFN)-γ and IL-6 were determined using commercial ELISA kits (MAGPIX™, Luminex®, MiraiBio, USA). The data were analyzed using the xPONENT® 4.2 software (MAGPIX™, Luminex®, MiraiBio, Alameda, CA). The standard curves used ranged from 1.95 to 32,000 pg/mL. IL-10 (Abcam, USA) was measured by a commercial ELISA kit, according to the manufacturer’s instructions. The final cytokine concentrations were normalized by the total protein concentration of each sample. Serum adiponectin levels were measured with a commercial ELISA kit (R&D System, USA).

### Chemotaxis assay

Spleen cells were dissociated as described for flow cytometry and 1.5 × 10^6^ cells in suspension in 1 mL of R10 medium with 10% fetal bovine serum (Millipore, USA) were added to the upper compartment of a transwell (6.5 mm diameter and 8 µm pore size; Millipore, USA). Eighty to a hundred mg of mPVAT from the respective animal were added in the lower compartment. As a control, in the lower compartment only R10 medium was added, or in the upper compartment PVAT without spleen cells was added. Transwell was incubated in a 5% CO_2_ environment at 37 °C, for 60 min. Cells that migrated to the lower compartment were quantified in a Neubauer chamber and normalized for the mPVAT mass. For each animal, the test was performed in duplicate.

### Statistical analysis

Prisma 6 0.0 (GraphPad Software Inc., USA) was used for data and statistical analysis. Data are expressed as mean ± standard error (SEM). Mean value differences between standard diet and HFD groups were compared with unpaired *t-test.* Two-way analysis of variance (ANOVA) followed by Bartlett’s test for homogeneity of variance and Tukey post hoc test for multiple comparisons were used to compare standard diet and HFD groups that were submitted to sham-surgery or splenectomy. The significance level was *P* < 0.05.

## Supplementary Information

Supplementary Information.

## Data Availability

The datasets generated during and/or analyzed during the current study are available from the corresponding author on a reasonable request.
