# Supplementary material for: Immune spleen cells attenuate the inflammatory profile of the mesenteric perivascular adipose tissue in obese mice
Source: Sci Rep. 2021 May 27;11:11153. doi: 10.1038/s41598-021-90600-0 (PMC8160359; doi:10.1038/s41598-021-90600-0)
Supplement: Supplementary file 1 — Supplementary Information. [file 41598_2021_90600_MOESM1_ESM.docx]

**Supplementary information**

**Immune spleen cells attenuate the inflammatory profile of the mesenteric perivascular adipose tissue in obese mice**

Renée de Nazaré Oliveira da Silva^a^, Rosangela Aparecida Santos-Eichler^a^, Carolina Dias^b^, Stephen Fernandes Rodrigues^a^, Dominik S. Skiba^c,e^, Richardt Gama Landgraf^d^, Maria Helena Catelli de Carvalho^a^, Tomasz Guzik^c^, Ricardo Ambrósio Fock^b^, Eliana Hiromi Akamine^a^

**Supplementary Table S1**

| **Supplementary Table S1** – Information on the antibodies used in flow cytometry | | | |
| --- | --- | --- | --- |
| **Antibody** | **Clone** | **Fluorochrome** | **Vendor (catalog number)** |
| CD3e | 145-2C11 | FITC | BD Pharmigen (553062) |
| CD4 | RM4-5 | APC | BD Pharmigen (553051) |
| CD8a | 53-6.7 | PE | BD Pharmigen (553033) |
| CD45R/B220 | RA3-6B2 | PE | BD Pharmigen (553090) |
| F4/80 | T45-2342 | APC | BD Pharmigen (566787) |
| CD11c | HL3 | PE | BD Pharmigen (557401) |
| CD11b | M1/70 | FITC | BD Pharmigen (553310) |
| CD206 | C068C2 | FITC | BioLegend (141704) |
| Ly-6G and Ly-6C (Gr-1) | RB6-8C5 | PE | BD Pharmigen (553128) |

**Supplementary Figure S1**


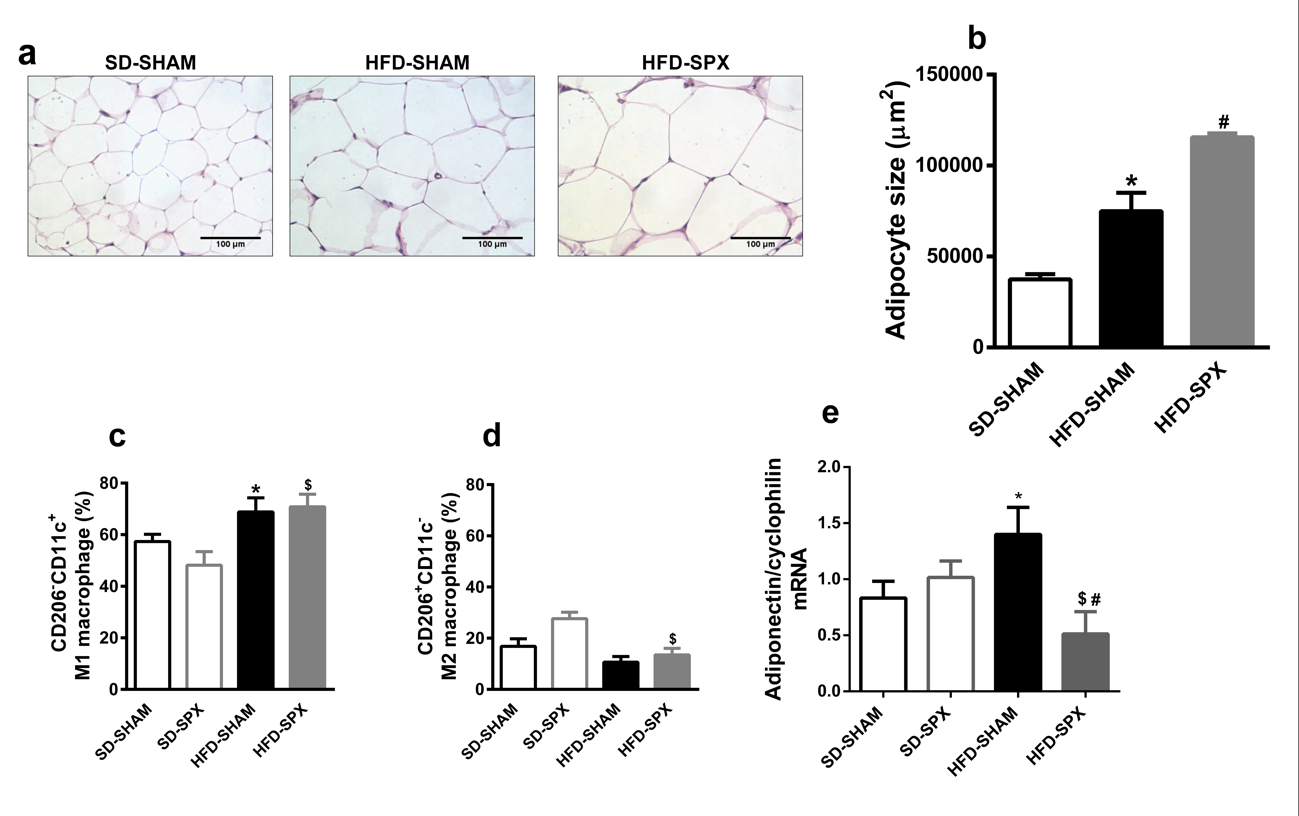


**Supplementary Figure S1 - Effect of splenectomy on morphology, M1 and M2 macrophages and adiponectin mRNA expression in periepididimal fat.** (a) Representative histological images and (b) adipocyte size of periepididymal fat of sham-operated (SHAM) mice fed standard diet (SD), SHAM mice fed high fat diet (HFD) and splenectomized (SPX) mice fed HFD. (n = 5 per group). Population of (c) M1 macrophages (identified as F4/80^+^ CD206^-^ CD11c^+^) and (d) M2 macrophages (identified as F4/80^+^ CD206^+^ CD11c^-^) in the periepididymal fat of SHAM and SPX mice fed SD or HFD. Results from two different experiments (n = 10 to 13 per group). (e) mRNA expression of adiponectin in periepididymal fat of SHAM and SPX mice fed SD or HFD. (n= 5 per group). Data were expressed as mean ± SEM. ANOVA: *P < 0.05, HFD-SHAM versus SD-SHAM; ^$^P < 0.05, HFD-SPX versus SD-SPX; ^#^P < 0.05, HFD-SPX versus HFD-SHAM.

**Supplementary Figure S2**

(a) TNF-α (17 kDa)

H

H

S

S

H

S

H


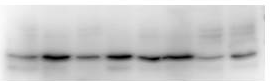


S

kDa

(b) Ponceau staining

50

H

S

H

S

H

S

H

S


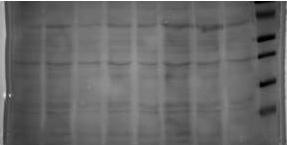


15

25

35

40

(c) IL-10 (15 kDa)

H


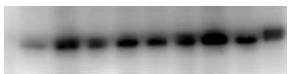


*

H

S

H

S

H

S

S

(d) Ponceau staining

kDa

H

S

H

S

H


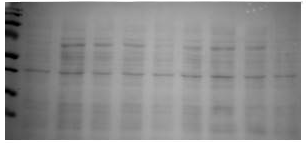


*

70

50

40

35

25

15

H

S

S

**Supplementary Figure S2 – Full-length blots shown in Figure 5.** Immunoblotting for (a) TNF-α detected at approximately 17 kDa and (b) the respective membrane stained with Ponceau. Immunoblotting for (c) IL-10 detected at approximately 15 kDa and (d) the respective membrane stained with Ponceau. S, mice fed with standard diet. H, mice fed with high fat diet. * sample loaded incorrectly.

**Supplementary Figure S3**

**
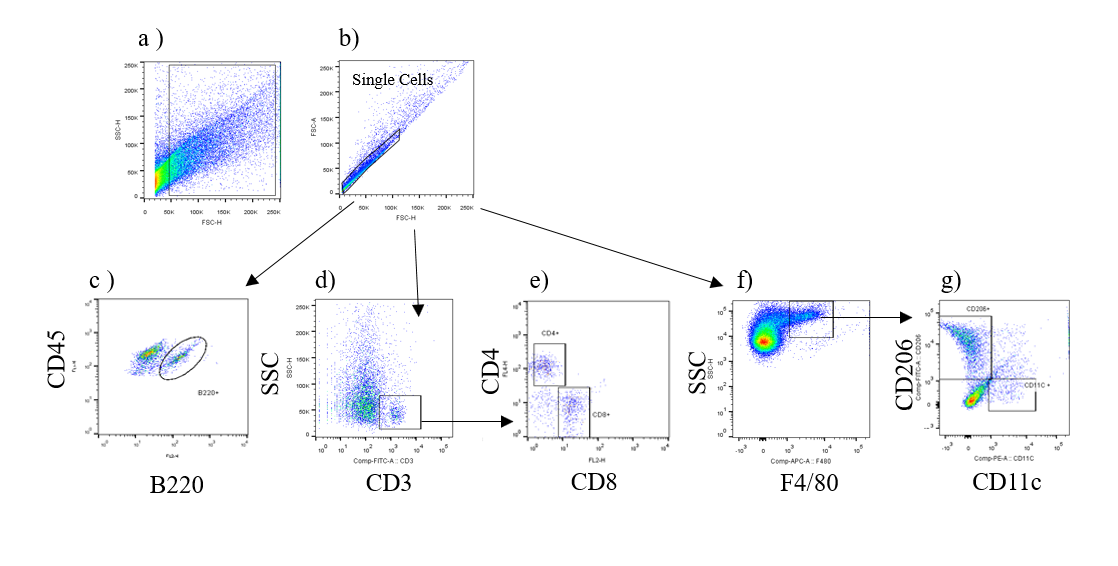
**

**Supplementary Figure S3 - Flow cytometry gates strategy for Figure 3.** a) Forward Scatter x Side Scatter, b) single cells c) CD45-FITC and B220-PE d) CD3+ -FITC e) CD3+ FITC CD4 +-APC or CD3 +FITC CD8 + PE f) F4/80+ APC f) F4/80+ APC CD206 + FITC or F4/80+ APC CD11c + PE.

**Supplementary Figure S4**

**
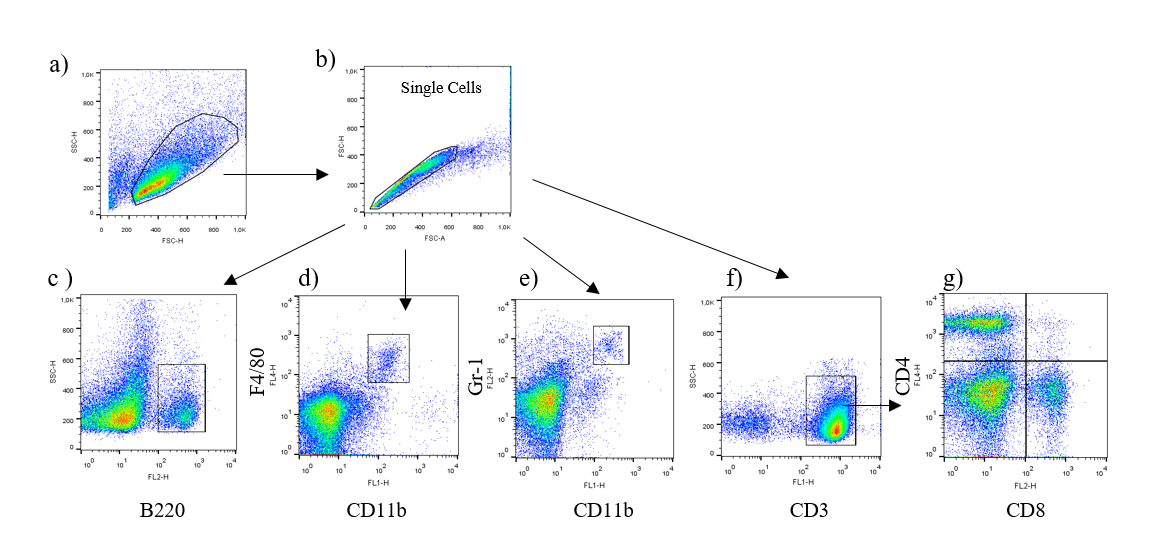
**

**Supplementary Figure S4 - Flow cytometry gates strategy for Figure 5.** a) Forward Scatter x Side Scatter, b) single cells and c) B220+ d) F4/80+ and CD11b+ e) Gr-1+- and CD11b+ f) CD3 + g) CD3 + CD4 + or CD3 + CD8 +.

**Supplementary Figure S5**

**
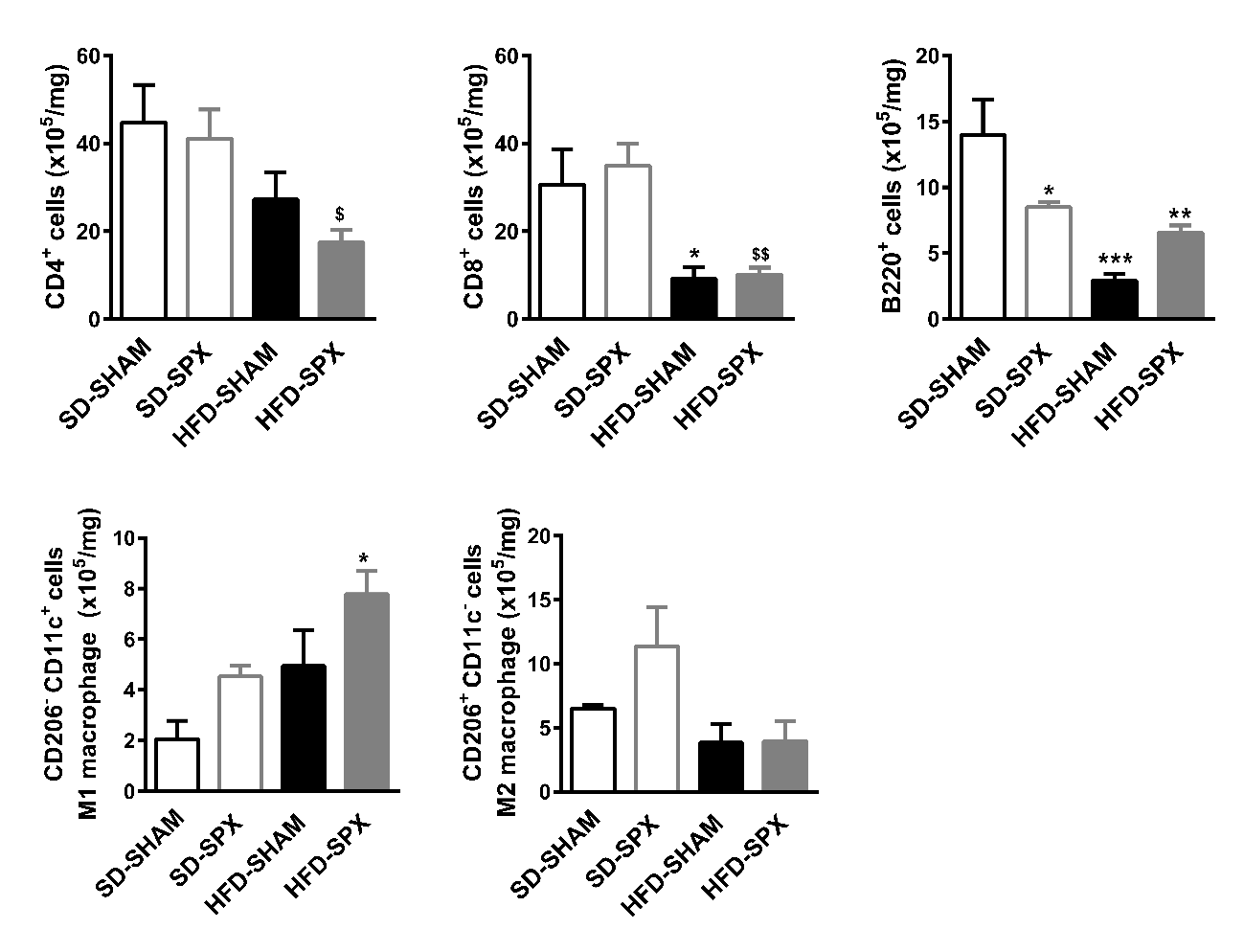
**

**Supplementary Figure S5 - Immunophenotyping of cells in mesenteric PVAT (mPVAT).** Absolute number of (a) CD4^+^ and (b) CD8^+^ T lymphocytes, (c) B220^+^ B lymphocytes and (d) CD206-CD11c^+^ (M1) and (e) CD206^+^CD11c^-^ (M2) macrophages in the mPVAT of sham-operated (SHAM) or splenectomized (SPX) mice fed with standard diet (SD) or high-fat diet (HFD). Results from two different experiments (n = 5 to 9 per group). Data were expressed as mean ± SEM. ANOVA: *P < 0.05, **P < 0.01 and ***P < 0.001, SD-SPX and HFD-SHAM versus SD-SHAM; ^$^P < 0.05, HFD-SPX versus SD-SPX.

**Supplementary Figure S6**

**
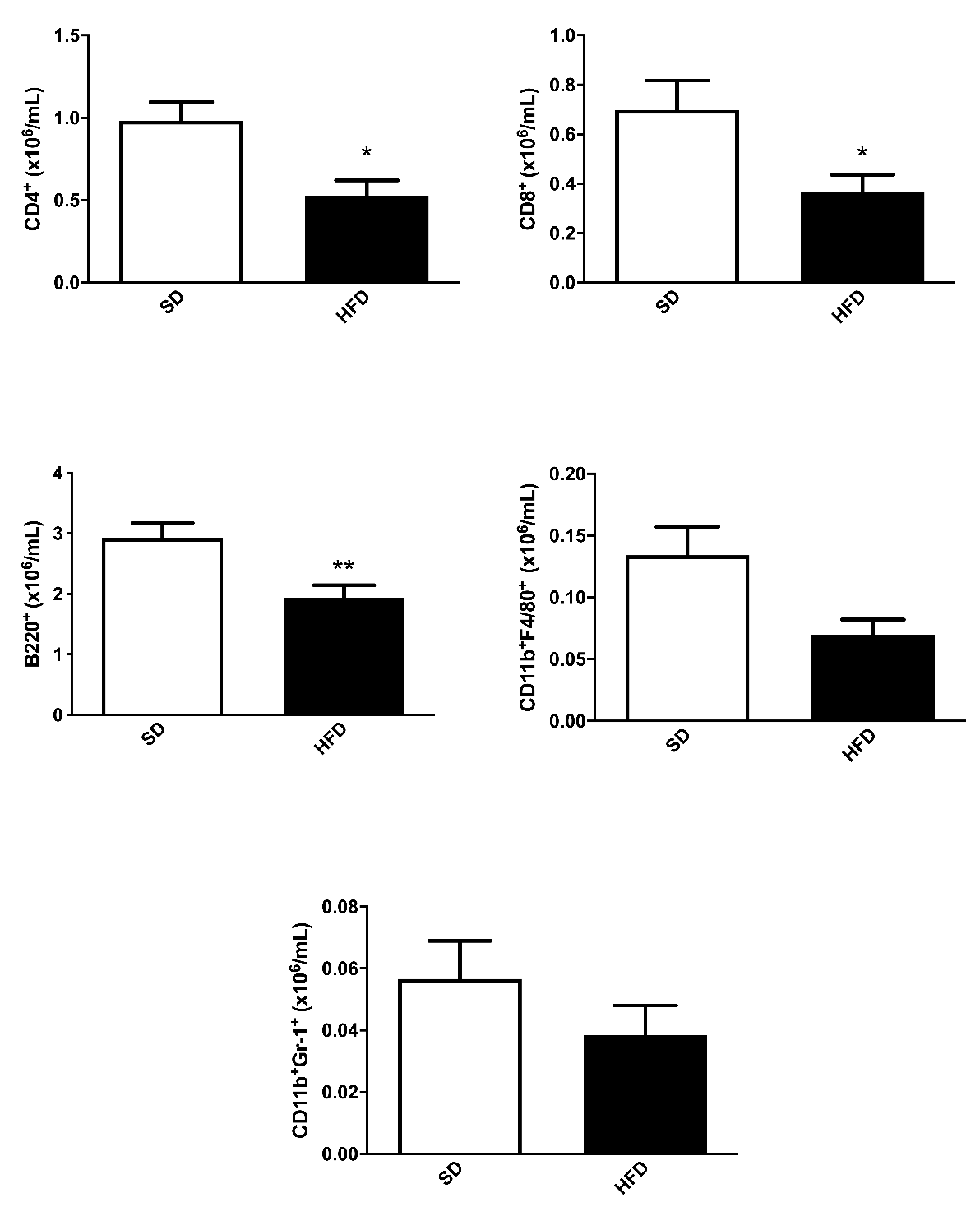
**

**Supplementary Figure S6 - Immunophenotyping of splenic cells.** Absolute numbers of (a) CD4^+^ and (b) CD8^+^ T lymphocytes, (c) B220^+^ B lymphocytes, (d) CD11b^+^ F4/80^+^ macrophages and (e) CD11b^+^ Gr-1^+^ granulocytes in the spleen of mice fed standard diet (SD) or high-fat diet (HFD). Results from two different experiments (n = 5 to 8 per group). Data were expressed as mean ± SEM. Unpaired t-test: *P < 0.05, HFD versus SD.
